# Cytopathologic Features of Metastatic Malignant Mesothelioma With SMARCB1 (INI‐1) Deficient Diagnosed by Ultrasound‐Guided Fine‐Needle Aspiration: A Case Report

**DOI:** 10.1002/dc.70073

**Published:** 2025-12-29

**Authors:** Jun Yang, Juandi Liu, Jing Jin, Deng Pan, Lei Shao

**Affiliations:** ^1^ Department of Cytopathology Ningbo Clinical Pathology Diagnosis Center Zhejiang China; ^2^ Department of Pathology Ningbo Medical Center Lihuili Hospital Zhejiang China; ^3^ Department of Experimental Pathology Ningbo Clinical Pathology Diagnosis Center Zhejiang China

## Abstract

Malignant mesothelioma (MM) is a rare yet aggressive neoplasm that arises from mesothelial cells lining the thoracic and abdominal cavities, the pericardium, and the tunica testis. Characterized by rapid progression, high invasiveness, and a poor prognosis, MM poses significant clinical challenges. SMARCB1, also referred to as INI‐1, hSNF5, or BAF47, is located on chromosome 22q11.2 and serves as a critical subunit of the SWI/SNF chromatin remodeling complex. INI‐1 functions as a tumor suppressor and plays a vital role in DNA damage repair and regulation of cell growth. Case Presentation: A 37‐year‐old Chinese male with no history of asbestos exposure but a 10‐year smoking history (30 cigarettes per day) presented with a cough lasting one month and a fever persisting for one day. Computed tomography (CT) showed multiple enlarged supraclavicular lymph nodes measuring up to 30 × 15 mm bilaterally. An ultrasound‐guided fine‐needle aspiration (FNA) of the left supraclavicular lymph nodes was subsequently performed. The SurePath‐prepared liquid‐based cytopathology (LBC) using Papanicolaou‐staining revealed tumor cells organized in clusters with indistinct borders. The cells exhibited abundant cytoplasm and pleomorphic round nuclei, which displayed prominent nucleoli and mitotic activity. Additionally, scattered lymphoid fragments were observed in the background. The FNA sample was formalin‐fixed and paraffin‐embedded for cell block; 4‐μm thick sections were prepared. Histological examination using HE‐stained cell block revealed a solid sheet‐like tumor growth characterized by minimal stroma and the absence of fibrovascular cores. The neoplastic cells had well‐defined borders, abundant eosinophilic (partially clear) cytoplasm, and round‐to‐oval nuclei displaying focal atypia, finely dispersed chromatin, and prominent nucleoli. Immunohistochemistry (IHC) demonstrated positive staining for CK(pan), calretinin, D2‐40, and SMARCA4, whereas NapsinA, TTF‐1, P40, NUT, and WT‐1 yielded negative results. The confirmed loss of INI‐1 expression ultimately led to the diagnosis of metastatic INI‐1 deficient MM. Conclusion: This case highlights the cytomorphological features and immunophenotype of INI‐1 deficient MM diagnosed through FNA. An accurate diagnosis requires a thorough clinicopathological correlation and a comprehensive IHC panel.

## 1 Introduction

Malignant mesothelioma (MM) is a rare but dreadful malignant tumor originating from mesothelial cells, lining the thoracic cavity, abdominal cavity, pericardium, and testicles. This disease is characterized by rapid progression, high invasiveness, and poor survival rates. According to the 5th World Health Organization (WHO) classification of thoracic tumors, MM is histologically classified into three subtypes: epithelioid (the most common and favorable prognosis accounting for approximately 50%–70% of cases), sarcomatoid (the most invasive and drug‐resistant subtype, accounting for about 10% of cases), and biphasic type (comprising approximately 30%–40% of cases) [1]. Current studies indicate that the majority of MM cases are attributable to long‐term asbestos exposure. In 2020, the WHO reported approximately 30,870 new cases of mesothelioma and 26,278 deaths attributed to this disease [2]. Previous research has shown that many patients are diagnosed with MM at advanced stages, for which clinical treatment options remain extremely limited, resulting in a 5‐year survival rate that ranges from 12 to 36 months [3].

The SWI/SNF chromatin remodeling complex functions as a tumor suppressor. This complex is composed of several subunits, including ARID1B, ARID1A, SMARCB1, SMARCA4, SMARCA2, and SMARCE1 [4]. While previous studies have predominantly focused on SMARCA4, which is frequently altered in thoracic neoplasms, there is considerably less information available regarding SMARCB1‐deficient neoplasms, which are rarely reported. SMARCB1, also known as INI‐1 (hSNF5/BAF47), is a core subunit of the SWI/SNF ATP‐dependent chromatin remodeling complex and is encoded on chromosome 22q11.2. INI‐1 is ubiquitously expressed in the nuclei of normal cells and can be identified through IHC staining [5]. Consistent with its role as a tumor suppressor, INI‐1 is crucial for various cellular functions, including DNA damage repair and the regulation of cell growth.

To date, there have been no reported cases of metastatic MM associated with SMARCB1 (INI‐1) deficiency identified through cytopathology. In this report, we present a case of metastatic MM characterized by INI‐1 deficiency diagnosed via a liquid‐based FNA sample for cytopathological examination and a cell block for HE and IHC ancillary tests.

## 2 Case Presentation

**FIGURE 1:**
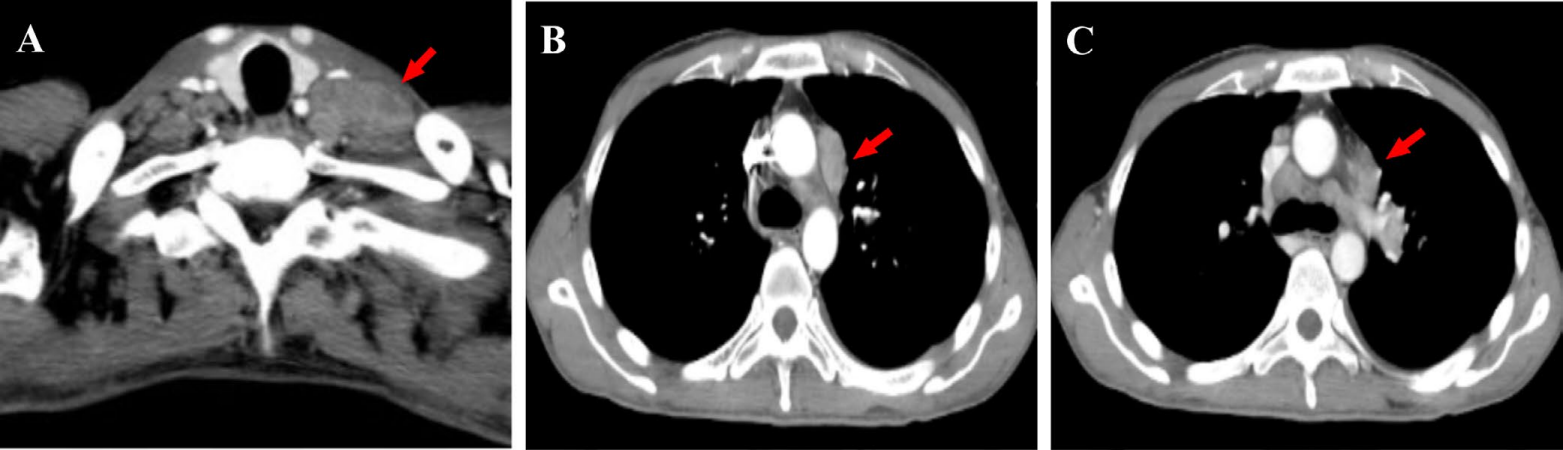
CT examination results. (A) The CT scan revealed the largest lymph node mass measuring approximately 30 × 15 mm located above the left clavicle (indicated by the red arrow); (B) A mass was observed adjacent to the aortic arch (indicated by the red arrow); (C) A mass was identified near the left hilar region of the lung (indicated by the red arrow).

The patient is a Chinese 37‐years‐old male with no history of asbestos exposure, but he has a 10‐year history of smoking consuming approximately 30 cigarettes per day, and he reports no alcohol consumption. He presented to the hospital with a cough lasting 1 month and a fever that persisted for 1 day. Laboratory investigations revealed elevated serum tumor markers indicative of pulmonary carcinoma: Ca‐125 at 6316.9 U/mL; and soluble cytokeratin fragment 19 (Cyfra21‐1) at 6.55 ng/mL. Other tumor markers, including CEA, SCC, and NSE, were within normal limits. CT scans showed multiple enlarged lymph nodes measuring up to approximately 30 × 15 mm located in the left hilum, mediastinum, adjacent to the aortic arch, and above the left clavicle, with some nodes demonstrating prominent annular enhancement on post‐contrast imaging (Figure 1). Ultrasound‐guided FNA of the left supraclavicular lymph nodes was performed, and the sample was sent for cytopathological examination. The FNA sample was fixed in formalin and the SurePath‐prepared liquid‐based smear was stained with Papanicolaou (PAP) stain. After fixation, the sample was centrifuged at 2500 rpm for 5 min. The supernatant was discarded, and the sediment was scooped out on filter paper. The sediment was processed routinely, similar to histopathological specimens. Paraffin‐embedded sections, 4 μm thick, were routinely stained with HE and IHC.

## 3 Cytopathology Features of the Liquid‐Based Cytology Findings

The SurePath‐prepared liquid‐based cytology (LBC) using PAP staining revealed tumor cells that form clusters distributed in patches, characterized by indistinct cellular borders. These cells exhibit abundant cytoplasm and predominantly round nuclei, which display marked pleomorphism. Prominent nucleoli are observed, and mitotic figures are evident. Additionally, scattered lymphoid fragments are present in the background.

On HE‐stained cell block sections, the histological features observed in FNA revealed that the tumor cells exhibited a solid, sheet‐like growth pattern with minimal stroma and an absence of a fibrovascular core. Certain tumor cells demonstrated abundant eosinophilic cytoplasm, eccentric nuclei, and prominent nucleoli, while some cytoplasm appeared transparent. The nuclei were round to oval in shape and exhibited significant focal atypia, characterized by fine, dispersed chromatin and visible nucleoli.

**FIGURE 2:**
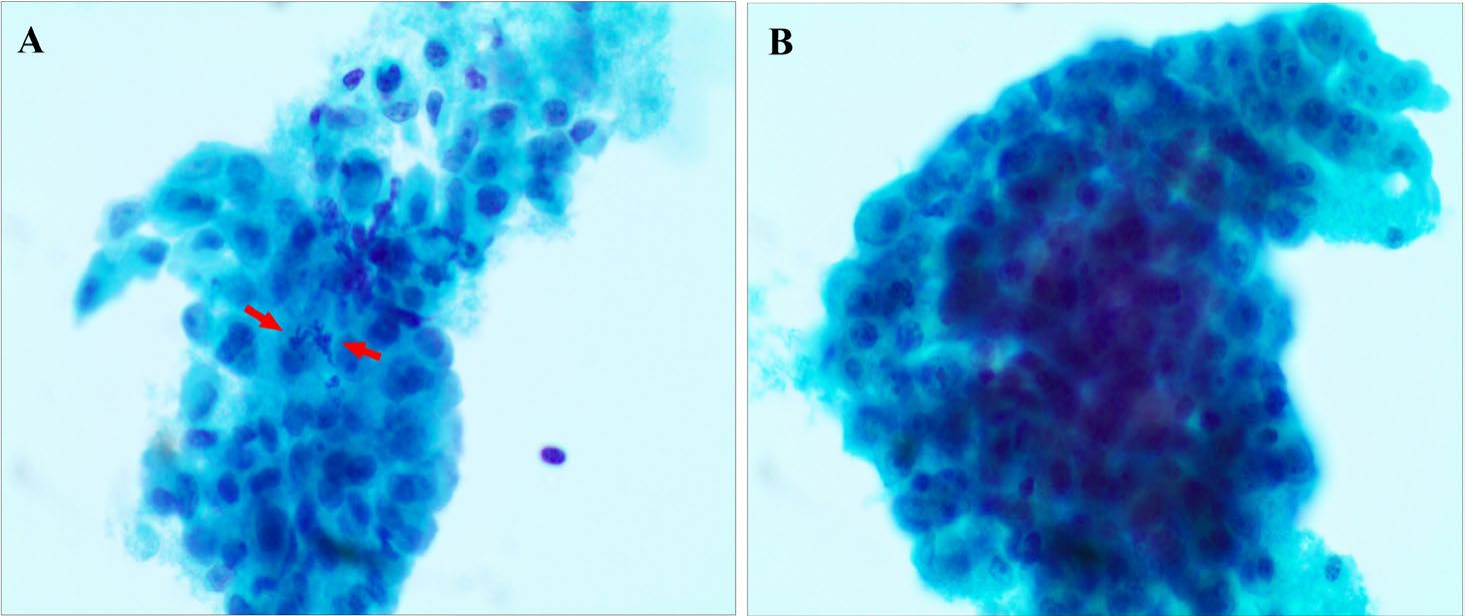
Photomicrographs illustrating cytohistological features reveal that tumor cells are organized into clusters that are distributed in patches. The tumor cells exhibit abundant cytoplasm and predominantly round nuclei, which display marked pleomorphism; atypical mitosis is observable (red arrow). (Papanicolaou‐stained, 400×).

**FIGURE 3:**
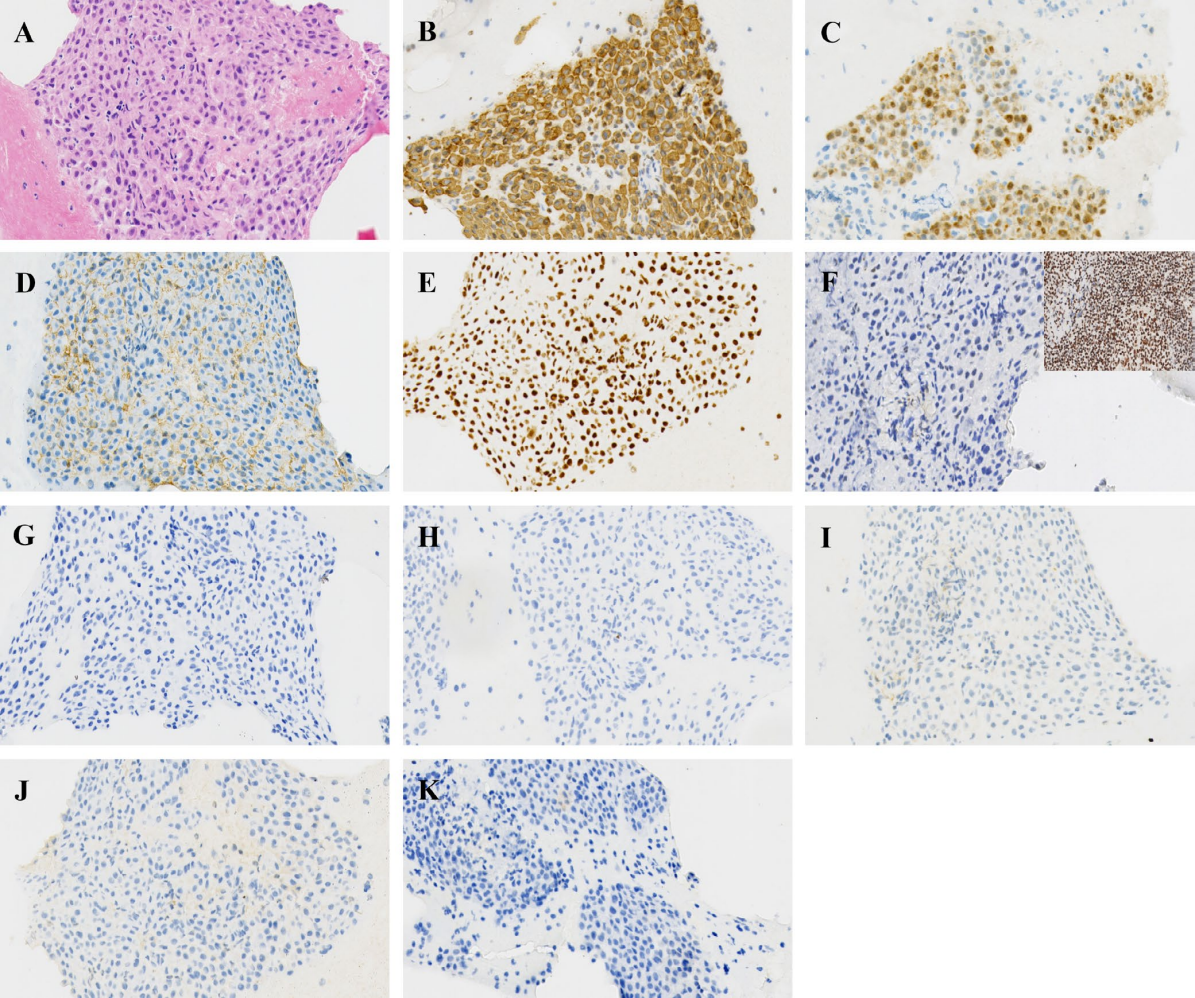
The histological features of FNA on HE‐stained cell block (A) and IHC staining. The tumor cells exhibit positivity for CK(pan) (B), calretinin (C), D2‐40 (D) and SMARCA4 (E), while showing negativity for INI‐1. The positive control image from tonsil tissue, which confirms INI‐1 expression is displayed in the upper right corner for reference (F). Additionally, the tumor cells are negative for NapsinA (G), TTF‐1 (H), P40 (I), NUT (J) and WT‐1 (K). (All images are at a magnification of ×200).

Immunohistochemical analysis shows that tumor cells are positive for CK(pan), calretinin, D2‐40, and SMARCA4, while they are negative for NapsinA, P40, TTF‐1, NUT, WT‐1, and demonstrate the loss of INI‐1 nuclear staining. The final diagnosis is metastatic MM with INI‐1 deficiency (Figures 2 and 3).

## 4 Discussion

The SWI/SNF chromatin‐remodeling complexes, also known as BRG1/BRM‐associated factor complexes, play a critical role in chromatin modification by regulating nucleosome positioning [4]. It has been demonstrated that SMARCB1/INI‐1, along with proteins involved in various pathways, such as the p16‐RB, WNT, and sonic hedgehog signaling pathways, are related to tumor proliferation, tumorigenesis, and progression [6].

In 1998, a study reported that positional cloning and sequence analysis revealed mutations, deletions, and other somatic changes in the SMARCB1/INI‐1 gene in malignant rhabdoid tumors [7]. Subsequently, aberrant expression of the INI‐1 protein has been documented in a variety of malignant tumors, including pediatric and adult mesenchymal tumors, such as sinonasal carcinoma [8, 9], gastrointestinal stromal tumors (GIST) [10], vulvar yolk sac tumor [11], pancreatic cancer [12], thyroid carcinoma [13] and intrathoracic neoplasms [14]. MM associated with INI‐1 deficiency are exceedingly rare.

In histopathology, MM with INI‐1 deficiency has been previously described in two studies. The first study illustrated that an INI‐1 deficient pleural MM with rhabdoid features was reported in 2017 by Kimura et al., a patient without asbestos exposure who died of a large amount of uncontrollable pleural effusion. Autopsy revealed the tumor had completely filled the left pleural cavity. Microscopically, the malignant cells exhibited rhabdoid features and IHC demonstrated that the tumor cells were positive for vimentin, D2‐40, BAP‐1, calretinin, CAM5.2 and AE1/AE3, while being negative for INI‐1, WT‐1, TTF‐1, CEA, Ber‐EP4, CK7, P40, CK20, desmin, CD34, BCL‐2 and S100, Additionally, fluorescence in situ hybridization (FISH) analysis showed tumor cell p16 deletion [15]. Another study found that 67% of deciduoid mesotheliomas exhibited a more frequent diminished expression of INI‐1 protein compared to 14% in epithelioid and 8% in biphasic types. Furthermore, this study highlighted that based on SMARCB1/INI1 expression, there were no statistically significant differences in survival between cases with reduced versus preserved expression [16].

In this paper, we present a case that underscores several key diagnostic considerations regarding INI‐1 deficient MM, particularly in the cytopathology of the FNA sample. Microscopic examination revealed cytomorphologic features characterized by cohesive cell clusters with indistinct borders, abundant cytoplasm, marked nuclear pleomorphism, prominent nucleoli, and mitotic activity, which are classic indicators of epithelioid mesothelioma. Notablly, the degree of atypia and pleomorphism observed in this case was strikingly pronounced. This observation is consistent with the established association between INI‐1 deficiency and tumors that exhibit rhabdoid or poorly differentiated morphology as previously reported by Kimura et al. [15]. Therefore, cytopathologists should consider the possibility of INI‐1 deficient neoplasms, including mesothelioma, when encountering high‐grade epithelioid features in metastatic regions, such as lymph nodes.

A panel of specific IHC markers on a cell block is essential for accurate final diagnosis. This case highlights the critical importance of cell block preparation from FNA samples. The diagnosis is established based on the characteristic immunoprofile, which includes the co‐expression of mesothelial markers such as CK (pan), calretinin, and D2‐40, alongside the loss of INI‐1 nuclear expression. The retention of SMARCA4 expression is pivotal in excluding other thoracic malignancies that are deficient in SMARCA4. The inactivation of epithelial markers, including TTF‐1, NapsinA, P40, and other specific markers such as NUT was vital in narrowing the differential diagnosis and ruling out primary pulmonary adenocarcinoma, squamous cell carcinoma, or NUT carcinoma metastasizing to lymph nodes. This immunophenotype aligns with previously reported histological cases of INI‐1 deficient MM [15, 16].

Although INI‐1 loss is rare, it has been documented in certain carcinomas, including sinonasal carcinoma and renal medullary carcinoma. In cases of INI‐1 deficient epithelioid malignancies involving supraclavicular lymph nodes, the primary differential diagnoses consist of metastatic poorly differentiated carcinomas, such as those originating from the lung, breast, or thyroid. Accurate diagnosis necessitates the use of lineage‐specific markers (TTF‐1, NapsinA, P40, Pax8) in conjunction with mesothelial markers (CK‐pan, calretinin, WT‐1, D2‐40). Epithelioid sarcoma typically exhibits INI‐1 loss and most commonly arises in soft tissue or lymph nodes. Immunohistochemically, these tumors frequently express cytokeratin (CK) and epithelial membrane antigen (EMA) but are generally negative for mesothelial markers such as calretinin, D2‐40, and WT‐1. CD34 positivity may be observed in some cases. In contrast, malignant rhabdoid tumor can occur at diverse anatomical sites, including soft tissue, and similarly demonstrate INI‐1 loss along with CK expression. However, they lack characteristic mesothelial immunomarkers, aiding in their distinction from other neoplasms. In our case, the tumor exhibited epithelioid morphology in both LBC and cell block preparations, which facilitated the differential diagnosis. While Kawai et al. reported a more frequent reduction of INI‐1 tumors with deciduoid morphology [16], our case displayed conventional epithelioid characteristic. Notably, the assessment of INI‐1 status should be a routine practice in atypical mesothelial proliferations. The comprehensive IHC panel applied to the cell block material was essential for systematically excluding alternative diagnoses and ultimately reaching a conclusive diagnosis of metastatic INI‐1 deficient MM.

The clinical and biological implications of INI‐1 deficiency in MM remain largely unexplored due to its rarity. Biologically, the loss of this core subunit of the SWI/SNF complex leads to widespread dysregulation of gene expression, typically associated with highly aggressive, poorly differentiated, or rhabdoid tumors across various anatomical sites [5, 6]. Clinically, in our case, the presentation with widespread lymph node metastasis in a young patient may reflect an inherently aggressive tumor biology, consistent with the behavior of INI‐1 deficient neoplasms. From a therapeutic perspective, the prognostic impact is still unclear. While one study found no statistically significant survival difference based on INI‐1 expression in mesothelioma [16], the aggressive features often linked to its loss warrant further investigation. Importantly, SMARCB1 deficiency can create unique therapeutic vulnerabilities. While not yet applied to mesothelioma, there is growing evidence that such tumors may be susceptible to targeted therapies, including EZH2 inhibitors, based on the concept of synthetic lethality, as well as immunotherapy due to their high tumor mutational burden and altered chromatin landscape [4, 6]. Therefore, identifying INI‐1 deficiency is not only of diagnostic value but may also open doors for future mechanism based therapeutic strategies, underscoring the importance of routine IHC screening in atypical or high‐grade mesotheliomas.

Our patient didn't have a history of asbestos exposure, which is consistent with previous study [15] and raises questions about alternative oncogenic mechanisms in INI‐1 deficient MM, potentially linked to smoking or other genetic alterations. The prognostic impact of INI‐1 loss in MM remains unclear, as Kawai et al. found no statistically significant difference [16]. The aggressive cytomorphology and advanced clinical presentation, including lymph node metastasis in our young patient, suggest a potentially aggressive tumor biology, which aligns with the adverse prognostic features of MM. This case underscores the diagnostic utility of FNA cytology combined with cell block immunophenotyping in rare malignancy subsets, such as INI‐1 deficient MM. Furthermore, it highlights the feasibility of detecting the critical protein loss of INI‐1 in cytologic specimens. For high‐grade epithelioid malignancies of uncertain origin, particularly those expressing mesothelial markers or arising in serosal‐associated sites, metastatic lymph node evaluation should include INI‐1 IHC to ensure diagnostic accuracy. Future studies should investigate larger cohorts of MM using IHC and molecular techniques to determine the prevalence of INI‐1 alterations.

## 5 Conclusion

This case highlights the cytomorphologic features and immunophenotypic profile of INI‐1 deficient MM diagnosed via FNA, thereby addressing a notable gap in the literature. Given the extreme rarity of this entity, a comprehensive diagnostic workup incorporating clinical findings, histomorphologic evaluation, cytomorphologic assessment, and a panel of key IHC biomarkers is essential for accurate diagnosis. From a cytopathologic perspective, this report expands the spectrum of INI‐1 deficient neoplasms, enhancing our diagnostic understanding and contributing to improved recognition of MM.

## Author Contributions

D.P. manuscript review. J.L. and J.J. collected clinical data and performed laboratory tests. J.Y. and L.S. collected the data and wrote the manuscript. All authors read and approved to submit the report for publication.

## Funding

This study was funded by the Medical and Health Research Project of Zhejiang Province (2022KY1186) and the Ningbo Top Medical and Health Research Program (2023010211).

## Ethics Statement

This study was approved by the Human Research Ethics Committee of Ningbo Clinical Pathology Diagnosis Center (NBPC‐LL‐SP1‐SYY02).

## Conflicts of Interest

The authors declare no conflicts of interest.

## Data Availability

The data that support the findings of this study are available from the corresponding author upon reasonable request.
